# Supplementary material for: Containment of a Multi-index B.1.1.7 Outbreak on a University Campus Through a Genomically Informed Public Health Response
Source: Open Forum Infect Dis. 2026 Apr 1;13(4):ofag096. doi: 10.1093/ofid/ofag096 (PMC13043073; doi:10.1093/ofid/ofag096)
Supplement: ofag096_Supplementary_Data [file ofag096_supplementary_data.zip › B.1.1.7_supplemental_figures.docx]

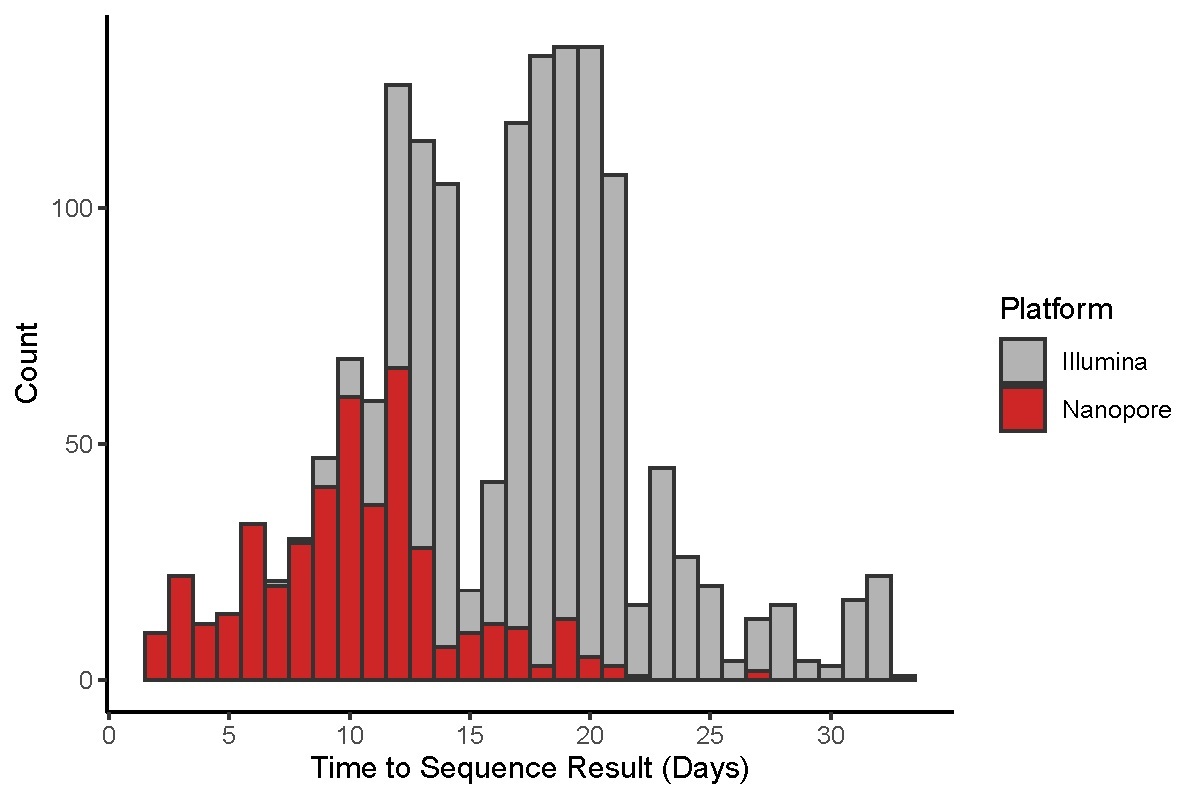


**Supplemental Figure 1.** Time between positive result and availability of sequence result.

Specimen count is plotted by turnaround time (number of days between positive result and completion of sequencing). Bars indicate specimens tested by Nanopore (red) and Illumina (gray) platforms.


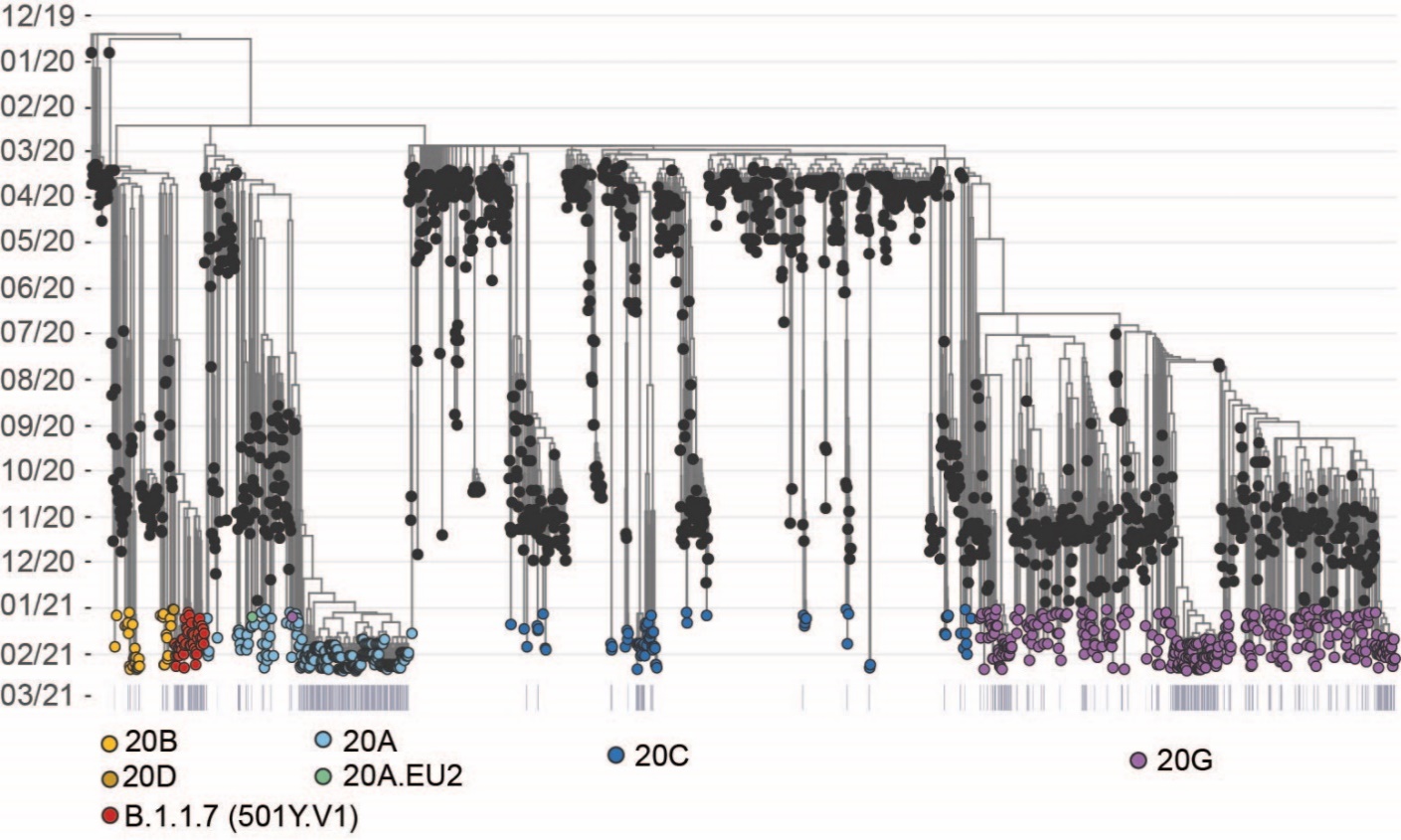


**Supplemental Figure 2.** Time calibrated maximum likelihood phylogenetic tree of all viruses sequenced from January through mid-February 2021 (colored tips, n=784). Of these 784 sequences, 436 were from students or campus-associated staff/faculty cases. Month is shown on the y-axis. Tips are colored by clade as follows: 20B (yellow), 20D (gold), B.1.1.7 (Nextstrain clade 501Y.V1, red, n=56), 20A (light blue), 20C (dark blue), 20G (purple). Contextual genomes have black tips (n=1682, not sequenced in our studies). Hashmarks below tree indicate which tips are from student cases. Accession numbers for all genomes used in this analysis are in Supplemental Table 3, inclusive of genomes sequenced as part of this study, which are also listed in Supplemental Table 4.


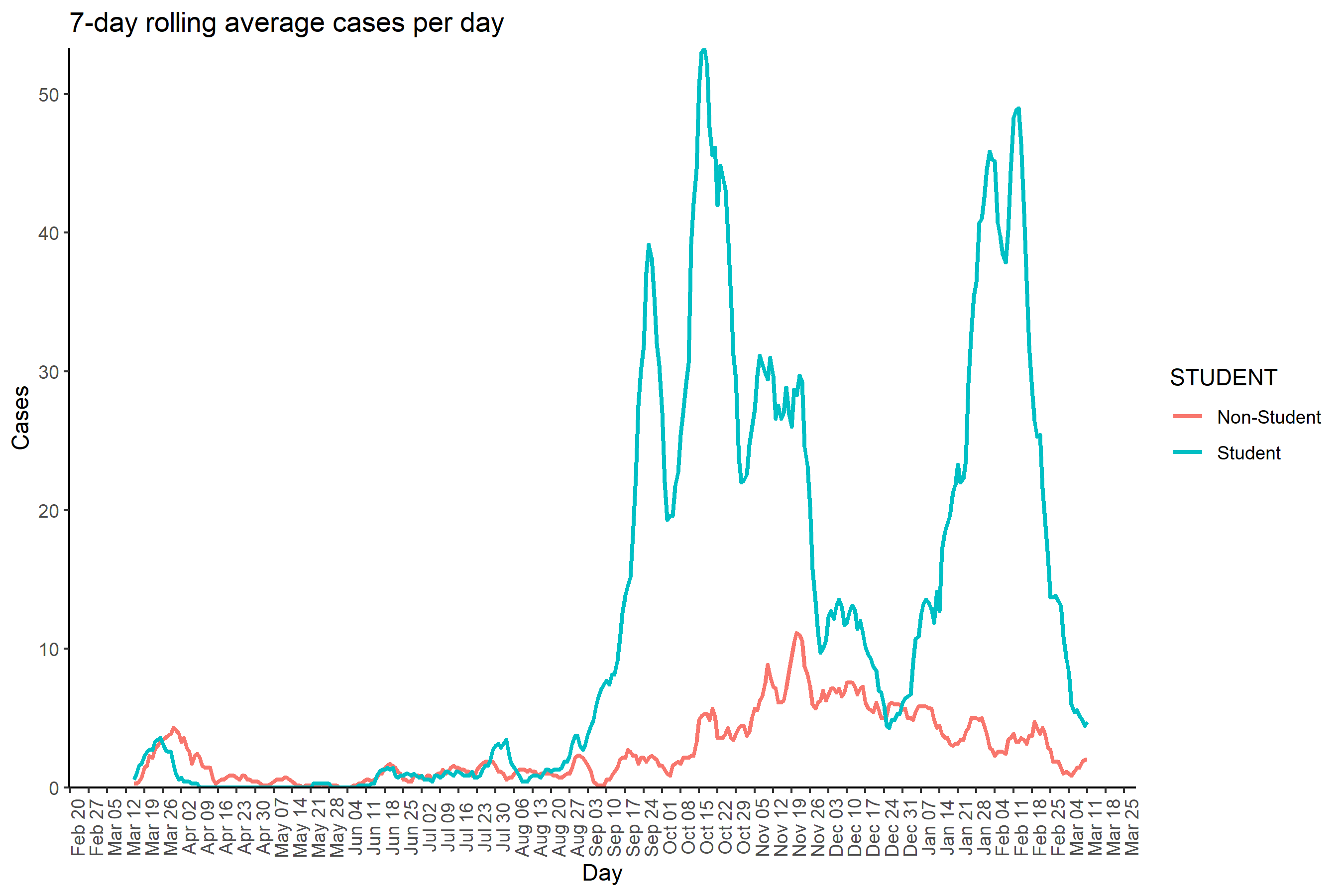


**Supplemental Figure 3.** Weekly totals of student and non-student cases identified for case investigation by university and local health departments.


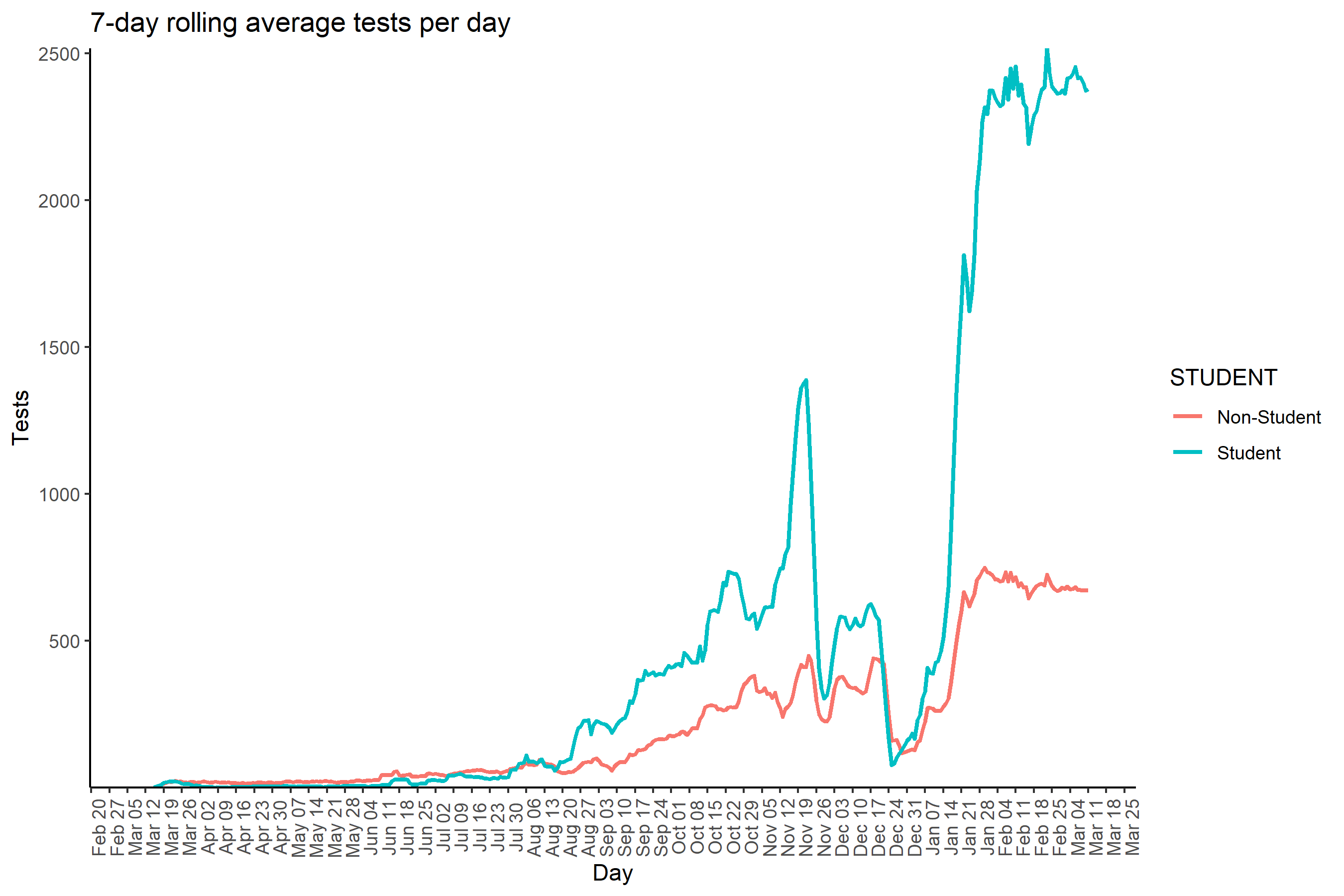


**Supplemental Figure 4.** Weekly totals of tests completed for asymptomatic and symptomatic individuals combined. The early January 2021 increase in testing coincides with return-to-campus asymptomatic testing conducted for students, faculty and staff.
